# Linking adolescents’ phubbing to depression: the serial mediating effects of peer relationship quality and psychological need frustration

**DOI:** 10.3389/fpubh.2024.1420151

**Published:** 2024-12-05

**Authors:** Heng Yue, Shiwen Gao, Fei Feng, Feiteng Wu, Hugejiletu Bao, Xuemin Zhang

**Affiliations:** ^1^School of Journalism and Communication, Xiamen University, Xiamen, China; ^2^College of Physical Education, Inner Mongolia Normal University, Hohhot, China; ^3^College of Business & Public Management, Wenzhou-Kean University, Wenzhou, China; ^4^Department of Physical Education, Xiamen University, Xiamen, China; ^5^School of Humanities and International Education, Baotou Medical College, Baotou, China

**Keywords:** phubbing, depression, peer relationship quality, psychological need frustration, mediation analysis

## Abstract

**Introduction:**

Phubbing is defined as a phenomenon in which individuals use their smartphones during conversations with others, focusing on the smartphones and escaping from interpersonal interactions. This phenomenon has been extensively studied in the literature. However, most studies concerning phubbing have focused on understanding its impact on the receivers; few have investigated the consequences of phubbing on the actors themselves. The present study aimed to explore the link between adolescents’ phubbing behavior and depression, focusing on the underlying psychological mechanisms.

**Methods:**

A total of 441 Chinese high school students participated in this study in September 2023. SPSS 25.0 and AMOS 24.0 were used to analyze the data. A hierarchical linear regression analysis was used to test the effect of phubbing on depression. A serial mediation model was applied to assess the roles of peer relationship quality and psychological need frustration in the link between phubbing and depression.

**Results:**

This study found that among adolescents, (1) phubbing had a significant direct association with depression; (2) phubbing had two significant indirect associations with depression, separately mediated via peer relationship quality and psychological need frustration; (3) the direct effect of phubbing on depression was sequentially mediated through peer relationship quality and psychological need frustration; (4) these results were applicable to both male and female adolescents.

**Discussion:**

These findings suggested that interventions aimed at improving peer relationships and addressing psychological need frustration might reduce depressive symptoms associated with phubbing in adolescents.

## 1 Introduction

With the development of science and technology, smartphones have been widely popularized in our daily lives. The portability offers people the opportunity to use smartphones to complete numerous tasks regardless of time and place; various applications also make people concentrate on them (1). Therefore, individuals may spend a large amount of time on their smartphones and develop bad usage habits. One of the problems caused by maladaptive smartphone usage is phubbing (2).

Conceptually, phubbing refers to the phenomenon that an individual avoids face-to-face interpersonal communication with others by focusing on the smartphone during a conversation without paying attention to the surroundings (3). Since phubbing has two main characteristics: excessive smartphone usage and frequently ignoring the conversation partner(s), this behavior, in some scholars’ opinions, is a kind of smartphone addiction and social exclusion (4, 5). Previous studies found that phubbing has various detrimental consequences, such as decreasing social interaction quality (6), lowering relationship satisfaction, and reducing relationship length (7). Besides, parental phubbing can lead to adolescents’ addictive behaviors, cyberbullying perpetration, social withdrawal, and aggression (8–10).

Although the adverse effects of phubbing have been extensively investigated, most of the previous studies focused on the impacts on the receivers; studies concentrated on the negative consequences on the actors themselves are relatively few (11, 12). Since both smartphone addiction and social exclusion can bring about various undesirable affects to the actors, such as psychological distress, shame and guilt, and lower levels of belonging (13–15). Phubbing, characterized by smartphone addiction and social exclusion, may also have unfavorable influences on the actors themselves. Since more than half of the Chinese middle school and high school adolescents stated that they are frequently distracted by smartphones when communicating with others (16), and pubbing is highly prevalent in this population (17). Therefore, the current study aimed to explore the effects of phubbing on adolescents, especially focusing on the association between phubbing and depression.

Apart from the main effect of phubbing on depression, potential mediators of this link are also required to be considered. On the one hand, the social displacement hypothesis posits that time spent on the internet usurps real-world social interactions and social activities, which may decrease the quality of interpersonal relationships and finally contribute to depression (18–20). As relationships with peers have become the center of adolescents’ lives (21), peer relationship quality may be a mediator in the link between phubbing and depression. On the other hand, the integrative model of mobile media use and need experiences (IM^3^UNE) assumes that psychological need frustration is a mediator of the association between mobile media use and well-being (ill-being) (22). Accordingly, phubbing may increase the level of depression through frustrating the actors’ psychological needs. In addition, since previous researchers have confirmed that low peer relationship quality can thwart the psychological need (23), peer relationship quality and psychological need frustration may play a mediating role in the association between phubbing and depression.

### 1.1 Phubbing and depression

Since phubbing is characterized by immoderate smartphone use, such as being immersed in smartphones and evading from interpersonal interactions, it has been considered a kind of smartphone addiction (5). Empirical studies have indicated that smartphone addiction directly increases the users’ severity of depression (11) and can also lead to loneliness, social anxiety, and low self-esteem, which indirectly leads to depression (13, 24, 25). Besides, the self-regulation model of depression posits that depression originates from the dysfunction of self-regulation (26). Previous studies have suggested that frequently engaging in phubbing may reduce self-regulation resources, decrease individuals’ self-regulation abilities, and inhibit the development of self-control (27, 28). Thus, it is possible that phubbing can positively predict the actors’ depression levels.

Besides, since phubbing often manifests dismissive behavioral characteristics, such as abruptly and frequently unprovoked disruptions of ongoing interactions accompanied by averted gaze and body posture, it may be perceived by their conversation partners as social exclusion (29). Empirical research suggests that people who socially exclude others may experience negative affect, guilt, and shame (14), and these negative feelings are antecedents of depression. Moreover, according to the interdependence theory, individuals’ behavioral and affective outcomes are influenced by their partners’ behavior (30). Since phubbing itself is a positive predictor of being excluded, in other words, the actors may also get excluded by the receivers (31). In this way, being socially rejected will hurt actors’ basic psychological needs; they may also have depressive feelings (32). Based on the perspectives of the two theories and the results of these empirical studies, the present study hypothesized that phubbing might increase the actors’ depression levels.

### 1.2 The mediating effect of peer relationship quality

Peer relationship quality is defined as the quality of social interactions among age-mates (33). Previous studies have indicated that phubbing receivers tend to perceive their conversation partners as less responsive and interact less intimately with them (34). As a result, the receivers’ may be less satisfied with their relationship (35). Since phubbing is interrelated between the two parties (36), the receivers’ undesirable experiences and problematic behaviors may reduce the actors’ perceived quality of peer interaction and peer relationships. Therefore, it is possible that people who exclude others tend to have lower levels of peer relationship quality. According to the interpersonal acceptance-rejection theory (37), phubbing behavior, as a kind of interpersonal rejection, may reduce the quality of peer relationships, which can further lead to hurtful emotions and increased levels of depression.

In terms of the association between peer relationship quality and depression, the vulnerability-stress model of psychopathology has indicated that poor interpersonal relationships may increase the possibility of depression (38, 39). Empirical studies have also documented that peer relationship quality is negatively associated with depression (40, 41). In these scholars’ opinion, individuals with low peer relationship quality will receive low levels of emotional support, have low degrees of self-efficacy and self-esteem, and these individuals cannot regulate their emotions effectively (42). All these detrimental effects may increase the severity of depression. Therefore, it is possible that peer relationship quality will significantly and negatively predict depression.

Besides, according to the social displacement hypothesis, the time spent on the smartphones reduces the time spent on the face-to-face social interactions; this may lower the quality of interpersonal relationships and subsequently bring about depression (18–20). In this way, phubbing, characterized by excessive smartphone use, may decrease the quality of adolescents’ peer relationships and then increase the severity of depression.

Based on the theoretical and empirical evidence, we hypothesized that phubbing behavior might reduce peer relationship quality, which in turn leads to increased depression in adolescents. In other words, peer relationship quality might mediate the link between phubbing and depression.

### 1.3 The mediating effect of psychological need frustration

Phubbing is a type of social exclusion (29). Empirical research indicated that social exclusion thwarts the actors’ autonomy and relatedness needs and subsequently leads to unpleasant affects (14). Accordingly, phubbing may directly frustrate the actors’ psychological needs and contribute to undesirable emotions. Besides, because the phubbing actor will be socially excluded by the receiver (31), in this way, the actor’s psychological needs may be thwarted by the exclusion of the receiver. Therefore, the receivers’ phubbing behaviors may frustrate the actors’ fundamental psychological needs and subsequently result in depression. In addition, previous studies have indicated that excessive smartphone use may provide social pressure for adolescents, decrease their self-control abilities, displace the time spent on real-life social activities, and hinder the development of social skills; all these consequences may thwart their autonomy, and relatedness needs (43). And engaging in social comparison through smartphones may also frustrate adolescent’s competence need (43). A recent longitudinal study has found that problematic smartphone use can significantly increase the risk of psychological needs frustration (44). Therefore, it is possible that phubbing may frustrate the actors’ psychological needs.

Depression is a mental disorder characterized by dysfunctional reactions to pressures and stressful situations (45). It may be most frequently associated with the loss of, difficulty in obtaining, or inability to achieve something one considers necessary for their perception of competence and value (45). Because people whose psychological needs are frustrated are often unable to think, feel, and act as they would like to, they may experience a sense of exclusion and social isolation, undergo more failures and inadequacies (46), and finally have higher levels of depression. Besides, some scholars have directly shown that psychological needs frustration can significantly enhance the severity of depression (47). Therefore, it was reasonable to infer that there existed a negative association between psychological need frustration and depression.

According to the IM^3^UNE, frequently engaging in phubbing may put the actors under pressure to check their smartphones, interrupt them from offline activities, and make them feel disconnected; these negative consequences can thwart their basic psychological needs and lead to depression (22). Based on this theory, the current study hypothesized that phubbing might frustrate the actors’ psychological needs in adolescents, enhancing their depression levels. In other words, psychological need frustration might mediate the link between phubbing and depression.

### 1.4 Serial mediating effects of peer relationship quality and psychological need frustration

The basic psychological need theory assumes that psychological need mediates the association between social context and individuals’ psychosocial adjustment; a need-thwarting environment may produce psychological need frustration, which can lead to psychopathology and ill-being (48). Some researchers have found that a low-quality interpersonal relationship may harm people’s psychological needs (23, 49, 50). Therefore, the present study hypothesizes that peer relationship quality can negatively predict psychological need frustration.

Based on the various empirical evidence and the arguments of the social displacement hypothesis and the IM^3^UNE, peer relationship and psychological need frustration may separately mediate the association between phubbing and depression; the basic psychological need theory also suggests there may be a negative association between peer relationship and psychological need frustration. Therefore, the current study assumes that there may exist a holistic relationship between these study variables. Possible process of this serial mediation model is that phubbing disturbs interpersonal interactions and makes victims feel offended; then the actors may have low-quality peer relationships, which may frustrate their psychological needs; consequently, they will experience depression. In other words, peer relationship quality and psychological need frustration might play serial mediating roles in the association between phubbing and depression. Theoretically, the present study might advance the understanding of phubbing by not only addressing its adverse impacts on the actors, but also identifying psychological mechanisms, such as peer relationship quality and psychological need frustration, that mediated this association. And practically, this study might contribute to drawing people’s attention to the adverse consequences of phubbing and subsequently changing the inappropriate smartphone usage behaviors.

### 1.5 Gender differences in the serial mediation model

There may exist gender differences in the serial mediation model. Previous studies have found that compared with males, females tend to spend more time on their smartphones to conduct various online activities, they may exhibit more phubbing behaviors (51–53). And since girls in adolescence are more sensitive to others affections and actions than boys, they often experience more peer problems and mental problems (54), which may in turn contribute to more psychological need frustration than their male counterparts (55, 56). Moreover, because the gender identity is intensified during adolescence, females may have more psychological distress (such as depression) than males (57). Nevertheless, the results of gender differences in these variables are inconsistent. Some studies showed there is no gender difference, or males tend to have higher levels of phubbing and depression (58–61). Other scholars also indicated the opposite results among adolescents by revealing that girls peer relationship quality is higher than boys, but their levels of psychological needs frustration are lower than the latter (62, 63). Due to these mixed evidences, any certain hypothesis would not be proposed. The present study would simply examine how the above serial mediating effects were influenced by gender.

### 1.6 The present study

**Figure 1:**
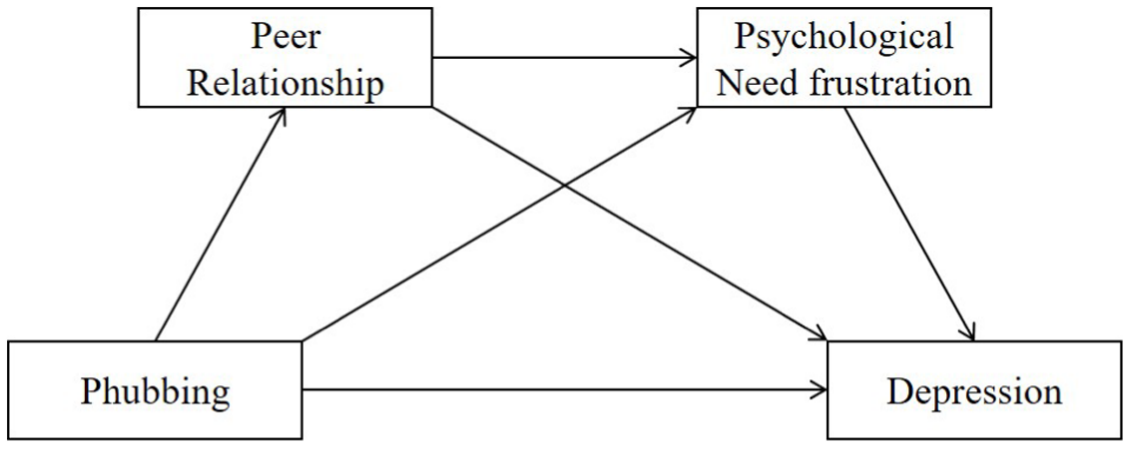
The serial mediation model.

In this study, a serial mediation model (Figure 1) was constructed to examine the association between phubbing and depression and the mediating effects of peer relationship quality and psychological need frustration in adolescents. There were four research hypotheses: (1) phubbing behavior would be positively associated with depression among adolescents; (2) peer relationship quality would mediate this relationship; (3) psychological need frustration would also serve as a mediator in this link; and (4) peer relationship quality and psychological need frustration would sequentially mediate the association between phubbing and depression.

## 2 Method

### 2.1 Participants

A paper-pencil-based survey was conducted to collect data. Participants were adolescents recruited from 5 high schools in China in the first 2 weeks of September 2023. After obtaining the consents from the students and their teachers, the survey was administered in a classroom setting during the class intervals or after class time; questionnaires were only sent to students who would like to participate in this study. The criteria for excluding invalid questionnaires were (1) containing incomplete responses, (2) having evident response patterns (e.g., selecting the same answer for all questions), and (3) choosing an incorrect option in the attention check items.

Initially, a total of 500 questionnaires were distributed; 479 were received. After removing 38 invalid questionnaires, the final sample included 441 adolescents. There were 251 males and 190 females. The mean age of the participants was 16.27 years old, ranging from 15 to 18 years.

### 2.2 Measures

Scales used in the present study were chosen based on their demonstrated reliability and validity in previous studies.

#### 2.2.1 Phubbing

The Phubbing Scale was used to assess phubbing behavior (3). This scale included 10 items; each was rated on a 5-point Likert scale, ranging from 1 (never) to 5 (always). An example item was: “My eyes start wandering on my phone when I’m together with others.” Higher scores on this scale reflected greater levels of phubbing behavior. In the present study, the Cronbach alpha coefficient was 0.839. The confirmatory factor analysis results suggested this scale had good construct validity: *χ*^2^ = 110.652, df = 33, RMSEA = 0.073, GFI = 0.952, CFI = 0.939, TLI = 0.917.

#### 2.2.2 Peer relationship quality

The Peer Relationships Scale was used to assess the quality of peer relationships (64). This scale included 15 items; each was rated on a 5-point Likert scale, ranging from 1 (never) to 5 (almost always). An example item was: “I was able to count on my friends.” Higher scores on this scale reflected higher-quality peer relationships. In the present study, the Cronbach alpha coefficient was 0.860. Items 1, 2, and 15 of the Peer Relationships Scale were removed due to low factor loadings (<0.40), following recommendations for maintaining construct validity (65). The scale retained good overall validity after these adjustments: *χ*^2^ = 148.964, df = 51, RMSEA = 0.066, GFI = 0.949, CFI = 0.939, TLI = 0.921. The following analyses only measured peer relationship quality by the remaining 12 items.

#### 2.2.3 Psychological need frustration

The Basic Psychological Need Frustration Scale was used to assess the level of psychological need frustration (66). This scale included 12 items; each was rated on a 5-point Likert scale, ranging from 1 (completely disagree) to 5 (completely agree). An example item was: “I feel forced to do many things I would not choose to do.” Higher scores on this scale reflected a greater level of psychological need frustration. The confirmatory factor analysis results indicated this scale had acceptable construct validity: *χ*^2^ = 156.07, df = 49, RMSEA = 0.07, GFI = 0.945, CFI = 0.934, TLI = 0.911. In the present study, the Cronbach alpha coefficient was 0.830.

#### 2.2.4 Depression

The Chinese version of the Patient Health Questionnaire (PHQ-9) was used to assess the severity of depression (67). This scale has been validated in adolescent populations, ensuring its appropriateness for this study (68). It included 9 items; each was rated on a 4-point Likert scale, ranging from 1 (not at all) to 4 (nearly every day). An example item was: “Over the last 2 weeks, how often have you thought that you would be better off or of hurting yourself in some way?.” Higher scores on this scale reflected greater levels of depression. In the present study, the Cronbach alpha coefficient was 0.828. The confirmatory factor analysis results demonstrated this scale had good construct validity: *χ*^2^ = 41.989, df = 27, RMSEA = 0.036, GFI = 0.979, CFI = 0.984, TLI = 0.979.

### 2.3 Statistical analyses

SPSS 25.0 was used for descriptive statistics, correlation analysis, and linear regression analysis. The mediating effects of peer relationship quality and psychological frustration were analyzed using the Amos 24.0. The goodness of the model fit was assessed using the following criteria: the ratio between *χ*^2^ and degrees of freedom (*χ*^2^/df) < 3, the root mean square error of approximation statistic (RMSEA) < 0.08, the comparative fit index (CFI) > 0.90, and the Tucker–Lewis Index (TLI) > 0.90 (69, 70). The mediating effects were considered significant if the 95% confidence intervals did not include zero, indicating a meaningful indirect effect (71).

## 3 Results

### 3.1 Common method bias test

The Harman’s single-factor test was conducted to test the possible common method bias. The unrotated exploratory factor analysis results demonstrated that 10 factors were extracted and explained 58.55% of the total variation. The first principal factor accounted for only 19.72% of the variance, indicating that common method variance is unlikely to have significantly influenced the results, thereby enhancing the robustness of the findings (72).

### 3.2 Descriptive statistics

The results of descriptive statistics and correlation analysis (see Table 1) indicated that the positive correlation between phubbing and both psychological need frustration (*r* = 0.279, *p* < 0.01) and depression (*r* = 0.474, *p* < 0.01) suggested that higher levels of phubbing were associated with greater psychological distress. Conversely, the negative correlation between phubbing and peer relationship quality (*r* = −0.219, *p* < 0.01) indicated that frequent phubbing might harm interpersonal relationships. Besides, the negative association between peer relationship quality and both psychological need frustration (*r* = −0.253, *p* < 0.01) and depression (*r* = −0.291, *p* < 0.01) implied that good relationships with peers might decrease the levels of psychological need frustration and depression. Psychological need frustration was positively correlated with depression (*r* = 0.441, *p* < 0.01), demonstrating that psychological need frustration might increase adolescents’ depressive experiences.

**Table 1:** Descriptive statistics and correlation analysis.

Variables	Cronbach’s *α*	*M*	*SD*	1	2	3	4
1 PHUB	0.839	21.091	7.597	1			
2 PEER	0.860	39.116	8.174	−0.219**	1		
3 FRU	0.830	33.992	8.009	0.279**	−0.253**	1	
4 DEP	0.828	16.836	5.354	0.474**	−0.291**	0.441**	1

PHUB, phubbing behavior; PEER, peer relationship quality; FRU, psychological need frustration; DEP, depression. **p < 0.01.

### 3.3 The effect of phubbing on depression

**Figure 2:**
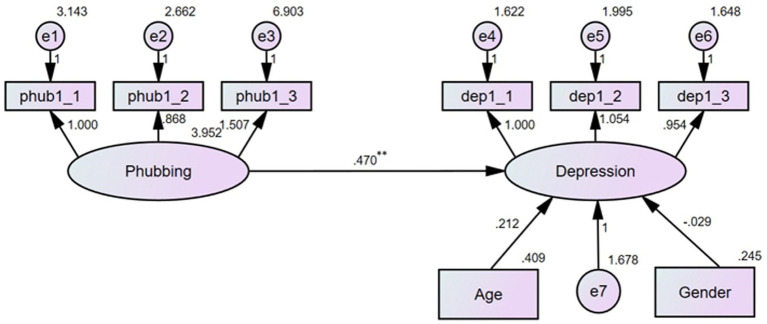
Results of the linear regression analysis (**p < 0.01).

To investigate the effect of phubbing on depression, a hierarchical linear regression analysis was performed (see Figure 2). The results showed that the model fitted the data well: *χ*^2^/df = 2.084, RMSEA = 0.050, CFI = 0.978, TLI = 0.968. After controlling for age and gender, phubbing significantly and positively predicted the level of depression (*b* = 0.470, *p* < 0.01), suggesting a moderate effect size, indicating that as phubbing behavior increases, so did the severity of depressive symptoms.

### 3.4 The mediating effect of peer relationship quality

**Figure 3:**
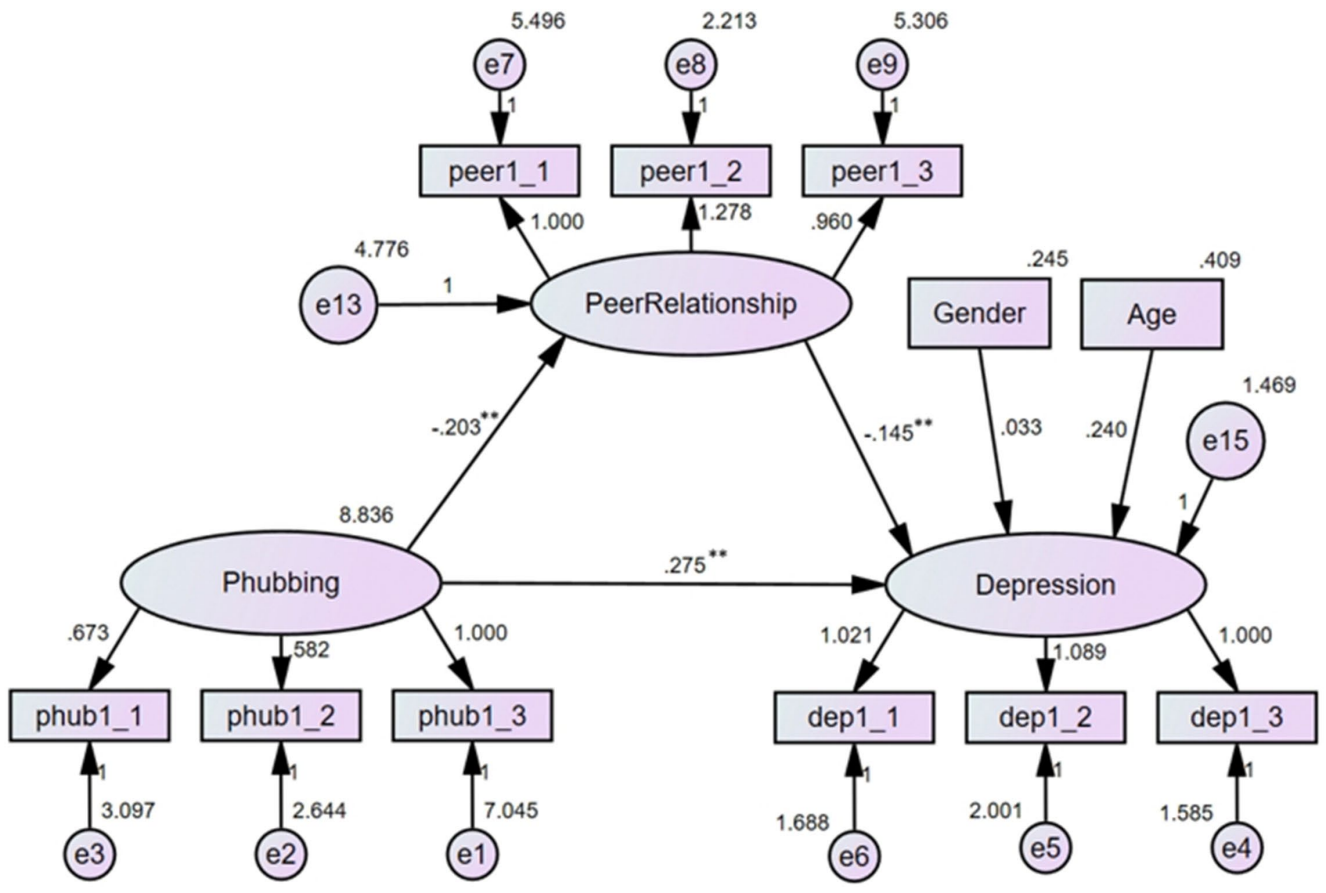
Mediating effect of peer relationship (**p < 0.01).

With phubbing as the independent variable, peer relationship quality as the mediator, and depression as the dependent variable, the mediating effect of peer relationship quality on the association between phubbing and depression was explored. The results (see Figure 3) indicated that the model fitted the data well: *χ*^2^/df = 1.941, RMSEA = 0.048, CFI = 0.973, TLI = 0.964. After controlling for age and gender, phubbing had a positively predictive effect on depression (*b* = 0.275, *p* < 0.01); this small effect size suggested that in this model, phubbing could increase the level of depression. Phubbing had a negatively predictive effect on peer relationship quality (*b* = −0.203, *p* < 0.01), which in turn could negatively predict depression (*b* = −0.145, *p* < 0.01). Although both the two effect sizes were small, they showed phubbing might decrease peer relationship quality, and the latter would elevate the severity of depression. The direct effect of phubbing on depression was significant (*effect size* = 0.275, 95% *CI* = [0.206, 0.354]), and the indirect effect through peer relationship quality was significant as well (*effect size* = 0.029, 95% *CI* = [0.011, 0.058]). Thus, peer relationship quality partially mediated the association between phubbing and depression, indicating that part of the impact of phubbing on depression operated through its negative effect on peer relationships. This suggested that interventions aimed at improving peer relationship quality could potentially mitigate some of the adverse effects of phubbing on mental health.

### 3.5 The mediating effect of psychological need frustration

**Figure 4:**
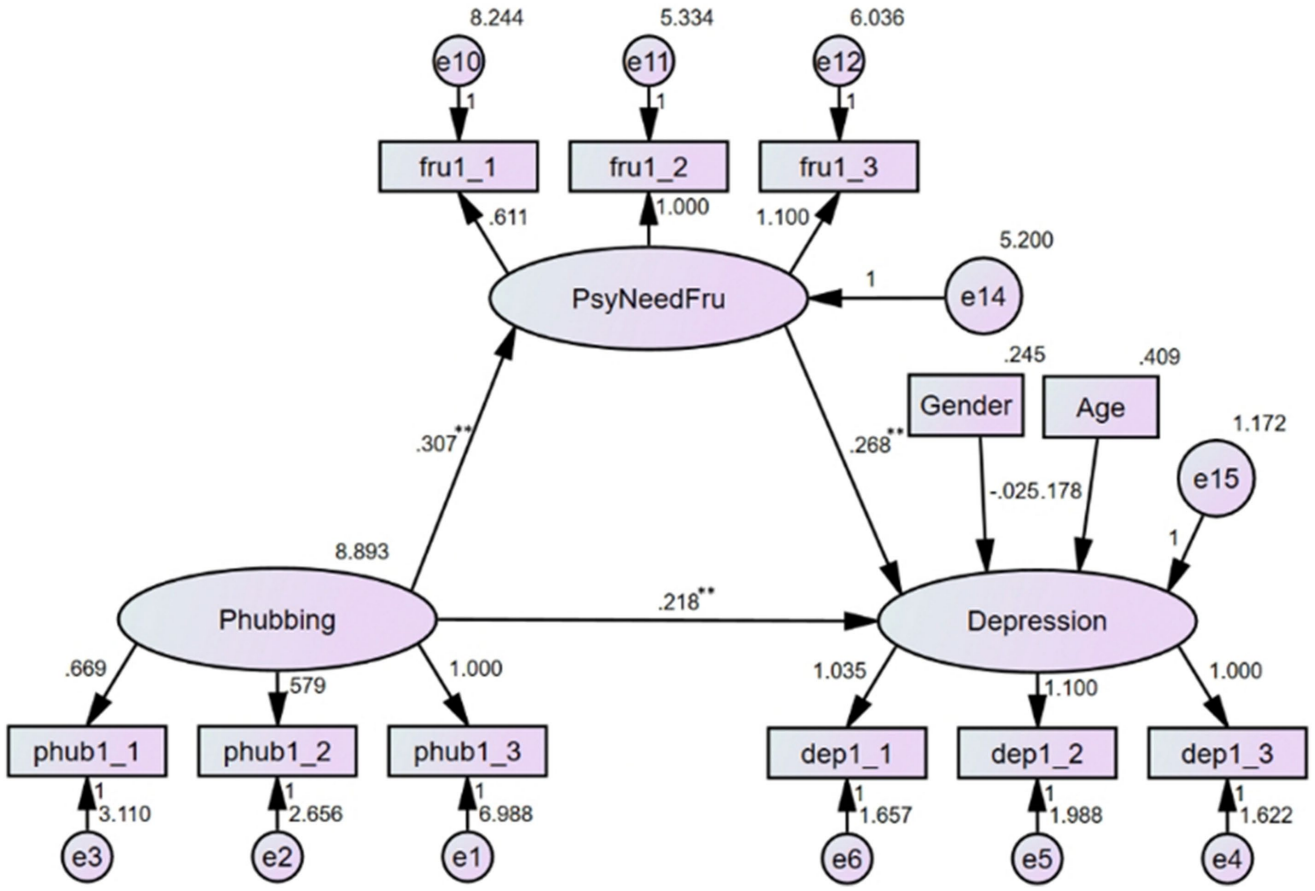
Mediating effect of psychological need frustration (**p < 0.01).

With phubbing as the independent variable, psychological need frustration as the mediator, and depression as the dependent variable, the mediating effect of psychological need frustration on the relationship between phubbing and depression was examined. The results (see Figure 4) indicated that the model fitted the data well: *χ*^2^/df = 1.597, RMSEA = 0.037, CFI = 0.981, TLI = 0.974. After controlling for age and gender, phubbing could positively predict depression (*b* = 0.218, *p* < 0.01), this small effect size suggested that in this model, the increment of phubbing behavior could bring about more depressive feelings. Phubbing positively predicted psychological need frustration (*b* = 0.307, *p* < 0.01), and psychological need frustration had a significant predictive effect on depression (*b* = 0.268, *p* < 0.01). The two small effect sizes demonstrated that phubbing might exacerbate psychological need frustration, the latter would aggravate depressive symptoms. The direct effect of phubbing on depression was significant (*effect size* = 0.218, 95% *CI* = [0.151, 0.292]), and the indirect effect through psychological need frustration was significant as well (*effect size* = 0.082, 95% *CI* = [0.050, 0.124]). Thus, psychological need frustration partially mediated the association between phubbing and depression, suggesting that phubbing might contribute to feelings of frustration related to unmet psychological needs, which in turn exacerbated depressive symptoms. This highlighted the importance of addressing psychological need frustration in interventions targeting phubbing-related depression.

### 3.6 Serial mediating effects of peer relationship quality and psychological need frustration

**Figure 5:**
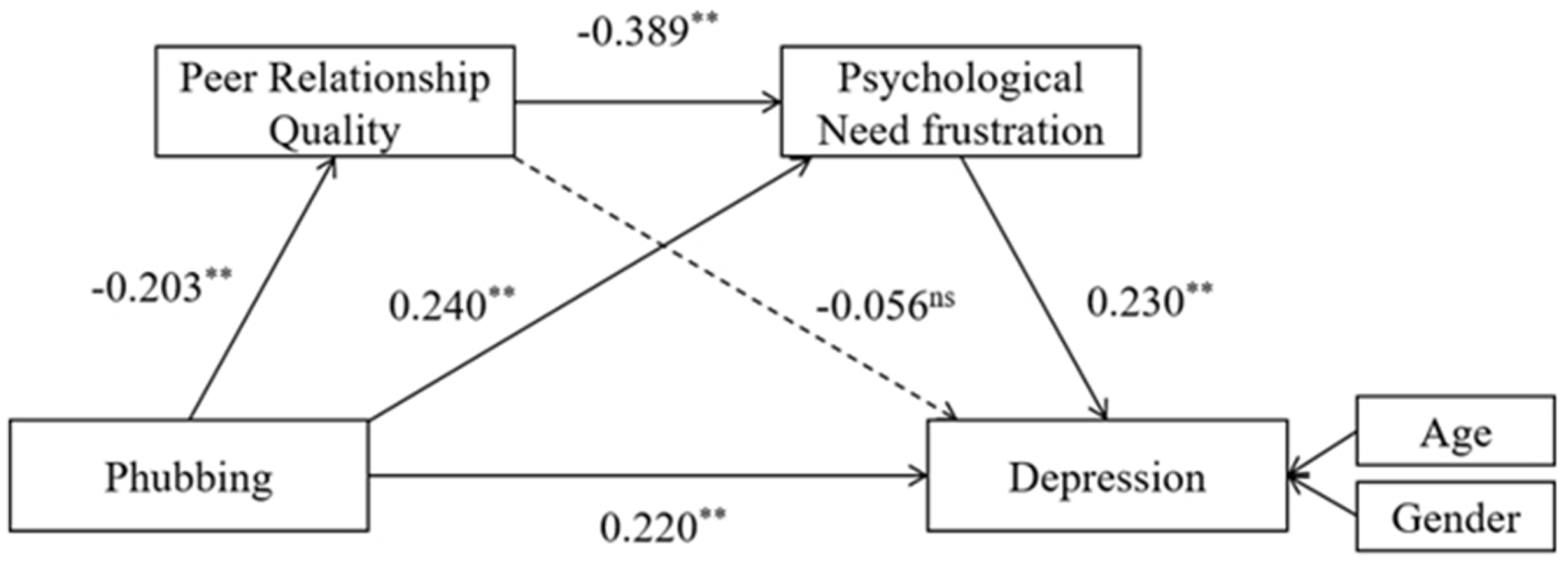
The results of the serial mediation model (**p < 0.01, ^ns^*p* > 0.05).

**Figure 6:**
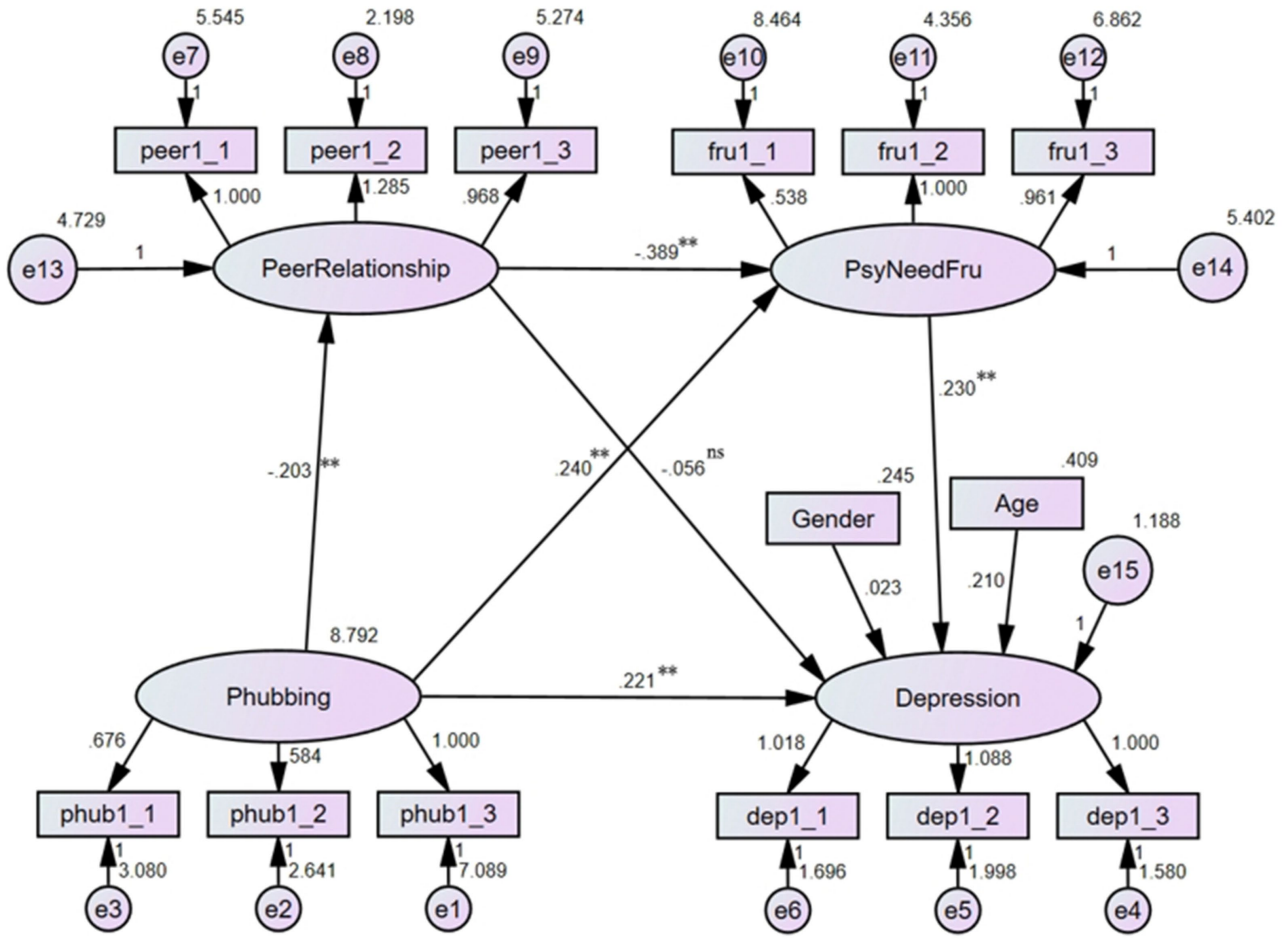
The results of the serial mediation model (**p < 0.01, ^ns^*p* > 0.05).

The serial mediation effects of peer relationship quality and psychological need frustration were tested (Figure 5). The results demonstrated that the model fitted the data well: *χ*^2^/df = 2.013, RMSEA = 0.048, CFI = 0.960, TLI = 0.949 (for more information, see Figure 6).

As seen, after controlling for age and gender, phubbing could negatively predict peer relationship quality (*b* = −0.203, *p* < 0.01), but positively predict psychological need frustration (*b* = 0.240, *p* < 0.01). Although both the two effect sizes were small, they indicated that phubbing would decrease peer relationship quality, but increase the level of psychological need frustration. Peer relationship quality had a significant negative predictive effect on psychological need frustration (*b* = −0.389, *p* < 0.01), this small effect size indicated that good peer relationship might relieve psychological need frustration. Phubbing significantly and positively predicted depression (*b* = 0.221, *p* < 0.01), peer relationship quality negatively predicted depression, but this link was not significant (*b* = −0.056, *p* > 0.05). The two small effect sizes suggested that as phubbing behavior increase, the severity of depression would go up, but the improvement in peer relationship quality could not directly relieve depressive feelings. Psychological need frustration had a significant positive predictive effect on depression (*b* = 0.230, *p* < 0.01), this small effect size implied the adverse impact of the former on the latter. The total direct effect of phubbing on depression was significant (*effect size* = 0.221, 95% *CI* = [0.152, 0.298]). The indirect effect of peer relationship quality was not significant (*effect size* = 0.011, 95% *CI* = [−0.005, 0.033]). The indirect effect of psychological need frustration was significant (*effect size* = 0.055, 95% *CI* = [0.027, 0.092]). The serial mediation effect of peer relationship quality and psychological need frustration was significant (*effect size* = 0.018, 95% *CI* = [0.008, 0.035]). The total indirect effect was significant (*effect size* = 0.085, 95% *CI* = [0.049, 0.129]). Thus, peer relationship quality and psychological need frustration sequentially mediated the link between phubbing and depression, although the direct effect from peer relationship quality to depression was not significant. These results revealed that both peer relationship quality and psychological need frustration contributed to the relationship between phubbing and depression. Specifically, the indirect effects suggested that phubbing led to depression both by diminishing the quality of peer relationships and frustrating psychological need. These findings underscored the multifaceted nature of phubbing’s impact on mental health, suggesting that effective interventions might need to address both social and psychological dimensions.

These findings collectively suggested that phubbing was a significant predictor of depression, with its effects being serially mediated by both peer relationship quality and psychological need frustration. This highlighted the need for holistic approaches in addressing the mental health impacts of phubbing, considering both social and psychological factors.

### 3.7 Gender differences in the serial mediation model

The gender difference of the serial mediation model was tested.

**Figure 7:**
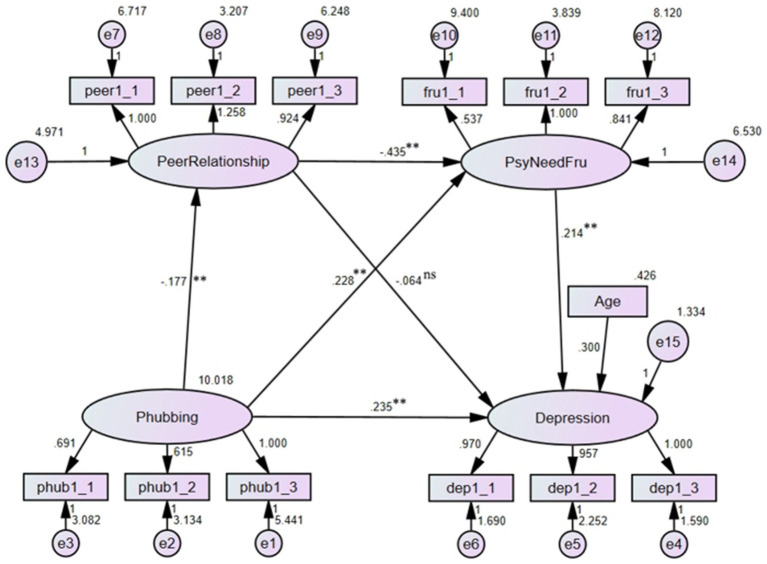
The results of the serial mediation model (Boys) (**p < 0.01, ^ns^*p* > 0.05).

In boys, the results indicated that the model showed a good fit to the data: *χ*^2^/df = 1.256, RMSEA = 0.032, CFI = 0.985, TLI = 0.980 (see Figure 7). The total direct effect of phubbing on depression was significant (*effect size* = 0.235, 95% *CI* = [0.156, 0.327]). The indirect effect of peer relationship quality was not significant (*effect size* = 0.011, 95% *CI* = [−0.007, 0.039]). The indirect effect of psychological need frustration was significant (*effect size* = 0.049, 95% *CI* = [0.016, 0.098]). The serial mediating effect of peer relationship quality and psychological need frustration were significant (*effect size* = 0.017, 95% *CI* = [0.005, 0.042]). The total indirect effect was significant (*effect size* = 0.077, 95% *CI* = [0.036, 0.132]).

**Figure 8:**
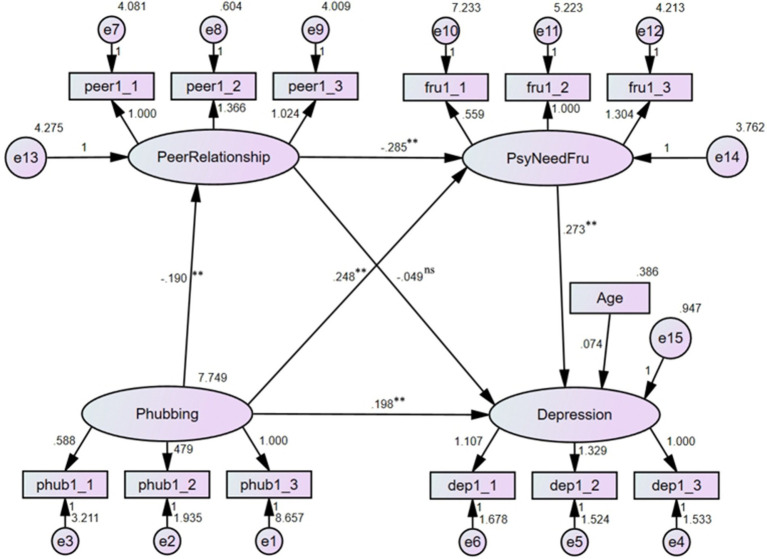
The results of the serial mediation model (Girls) (**p < 0.01, ^ns^*p* > 0.05).

In girls, the results indicated that the model offered a good fit to the data: *χ*^2^/df = 1.886, RMSEA = 0.068, CFI = 0.938, TLI = 0.917 (see Figure 8). The total direct effect of phubbing on depression was significant (*effect size* = 0.198, 95% *CI* = [0.075, 0.334]). The indirect effect of peer relationship quality was not significant (*effect size* = 0.009, 95% *CI* = [−0.008, 0.051]). The indirect effect of psychological need frustration was significant (*effect size* = 0.068, 95% *CI* = [0.026, 0.144]). The serial mediating effect of peer relationship quality and psychological need frustration were significant (*effect size* = 0.015, 95% *CI* = [0.002, 0.051]). The total indirect effect was significant (*effect size* = 0.092, 95% *CI* = [0.044, 0.187]).

A multi-group comparison analysis was also performed to examine the difference of the regression coefficients between male and female groups. The results indicated that the unconstrained model fitted the data well: *χ*^2^/df = 1.652, RMSEA = 0.039, CFI = 0.966, TLI = 0.953. Then, all the structural paths were constrained to be equal, the results showed that the model fitted the data well: *χ*^2^/df = 1.586, RMSEA = 0.037, CFI = 0.965, TLI = 0.957. The results of the comparison between the two models demonstrated that: Δ*χ*^2^(14) = 15.848, *p =* 0.323 (>0.05), ΔRMSEA = 0.002, ΔCFI = 0.001, ΔTLI = 0.004. According to the suggested cut-off values of these indices (73), there was no significant difference between the two gender groups, indicating that the path coefficients were similar for both genders.

## 4 Discussion

The primary purposes of the current study were to investigate whether there was an association between phubbing and depression in adolescents and whether peer relationship quality and psychological need frustration were mediators of this relationship. The key findings and implications were presented and discussed in the following sections:

### 4.1 The relationship between phubbing and depression

In line with previous studies revealing that there is a positive association between the two variables (3, 11), by focusing on the negative consequences of phubbing on the actors themselves, the findings of the current study indicated that phubbing could increase the actors’ depression levels. The reasons might lie in the following three aspects: Firstly, phubbing actors often prefer the virtual world instead of real-life ones; they tend to use smartphones to isolate themselves from the social environments (5). This will hinder them from acquiring the necessary social skills, damage their social relations, push them to life-threatening occasions, and decrease their satisfaction with life. All these negative influences will make phubbing actors experience more loneliness, anxiety, and depression (74). Secondly, phubbing was a type of social exclusion (4), and empirical studies confirmed that individuals who excluded others may experience greater negative affects (14, 75), feel self-depleted (76), and have lower levels of desire to establish new social relationships (77). Besides, phubbing itself is a risk factor of being neglected by others. Thus, whether an individual is considered the receiver or the actor of phubbing, these detrimental consequences would increase their depression levels. Finally, previous studies demonstrated that phubbing is a positive predictor of smartphone addiction; the latter can decrease the actors’ self-esteem, self-efficacy, perceived social support, and sleep quality, and then enhance the severity of depression (78, 79). From what had been mentioned above, the association between phubbing and depression could be understood. This study not only corroborated previous findings on the detrimental effects of phubbing but also extended the understanding by focusing on the actors themselves, highlighting the self-inflicted nature of these negative outcomes.

### 4.2 The mediating effect of peer relationship quality

The results of the current study suggested that peer relationship quality mediated the association between phubbing and depression.

For the first path of this mediation model, this study indicated phubbing was negatively correlated with peer relationship quality. Firstly, previous scholars have found that phubbing interrupts social face-to-face interaction; the receivers may perceive their interaction as rude and inattentive, feel annoying, disrespected, and socially excluded (80). These negative consequences may decrease the communication quality and contribute to the reduction of relationship quality. Secondly, phubbing others through smartphones may result in such behaviors being reciprocated unintentionally or purposefully (31), that is to say, phubbing actors might suffer from phubbing by the prior victims. According to the interpersonal acceptance-rejection theory, phubbing can be considered a kind of interpersonal rejection, which contributes to lower friendship satisfaction levels (81). Thus, after being socially excluded by the victims, the actors would be less satisfied with their peer relationships and report low levels of relationship quality. Thirdly, phubbing itself is a kind of smartphone addiction, which will damage their normal social functioning and interpersonal trust (82). These evidences might be helpful for understanding the negative association between phubbing and peer relationship quality.

For the second path of this model, this study demonstrated peer relationship quality was negatively correlated with depression. This was consistent with prior studies that indicate a negative association between the two variables (40). This might be because social relationships have been confirmed to be a key predictor of people’s mental health (83), and often, the loss of an important relationship is an important antecedent of depression (45). Therefore, the low-quality peer relationship resulted from phubbing may increase phubbing actors’ depression levels. According to the attachment theory (84), adolescents with high-quality peer relationships can obtain more social support from the significant people around them (85); the availability of peer relationships can serve as a coping mechanism for pressures and stressful situations (45), which may also protect them from depression.

To sum up, the mediating effect of peer relationship quality in the association between phubbing and depression could be understood. And this indicated that interventions aimed at enhancing social skills and peer interactions could be particularly effective in reducing depressive symptoms in adolescents who engage in phubbing.

### 4.3 The mediating effect of psychological need frustration

For the first path of this mediation model, the results of the present study indicated that phubbing was positively correlated with psychological need frustration. The reason might lie in the following aspects: Firstly, phubbing has been considered a form of social exclusion (4). Prior scholars revealed that socially excluding others has costs, such as thwarting the actor’s psychological needs for autonomy and relatedness (14), bringing the actor lower mood and belonging feelings, and making them perceive fewer connections with others (86). Therefore, phubbing might have similar negative impacts on psychological need frustration. Secondly, according to the need-threat model (87), social exclusion can negatively impact human needs. Thus, when phubbing actors may suffer from psychological need frustration as well when they were treated in the same way by the prior receivers. Thirdly, prior studies have confirmed that smartphone addiction can decrease individuals’ self-esteem (the need for self-esteem) and self-control (the need for self-control), make people experience more loneliness (the need to belong), and less life meaning (the need for meaningful existence) (88). By the same way, phubbing, a form of smartphone addiction, will also frustrate the actors’ basic psychological needs in these aspects. Therefore, the association between phubbing and psychological need frustration could be understood.

For the second path of this mediation model, the positive association between psychological need frustration and depression was confirmed in the present study. This result was supported by previous studies that demonstrated individuals whose basic psychological needs are frustrated often report more symptoms of depression and anxiety and less satisfaction with their lives (89). On the one hand, from the perspective of basic psychological need theory, satisfying psychological requirements is critical for an individual’s well-being and mental health. If individuals’ autonomy needs are frustrated, they may experience pressure and coercion; if their competence needs are frustrated, they may experience inadequacy and failure; and if their relatedness needs are frustrated, they may perceive loneliness and social alienation. Consequently, these negative impacts will make them more likely to have internalizing problems such as depression (60, 89). On the other hand, psychological need frustration can be considered a process in which people evaluate the impacts that the social conditions hinder their psychological needs (66). According to the information theory of emotion (90), negative emotions come from the shortage of the available information for solving problems. When individuals lack the necessary information to satisfy these needs (psychological needs are frustrated), they will have psychological distress, such as loneliness, anxiety, and depression.

All the empirical and theoretical evidence might help understand the mediating effect of psychological need frustration in the association between phubbing and depression. Future research could explore other potential factors, such as self-esteem or coping strategies, that might also influence the relationship between phubbing and depression.

### 4.4 Serial mediating effects of peer relationship quality and psychological need frustration

The results of the data analyses demonstrated a negative relationship between peer relationship quality and psychological need frustration. This might be because peer relationship is an important factor for adolescents’ mental development. According to the basic psychological need theory, low-quality peer relationship may serve as an adverse social-environmental factor that can thwart individuals’ psychological needs and lead to psychological problems (48). Empirical studies also indicated that a poor peer relationship may decrease adolescents’ self-efficacy, self-esteem, and self-control (91), hinder them from getting necessary life skills and social support, form interpersonal attachment and interpersonal trust (21), and contribute to loneliness, social anxiety, and other negative affects (92, 93). Consequently, their psychological needs, such as autonomy, competence, and relatedness needs, will be frustrated.

It is noteworthy that although peer relationship quality could significantly predict depression when it was individually added as the mediator, this link in the serial mediation model was not significant. It was possible that the effect of peer relationship quality on depression was mediated by psychological need frustration. According to the cognitive appraisal theory (94), emotions originated from individuals’ evaluations of their situations; cognitive appraisal is the central construct that mediates the association between the emotional response and the environment. Because psychological need frustration is a process in which people assess the threat effects of the social environments on their psychological needs, it may mediate the association between peer relationship quality and depression. Besides, according to the basic psychological need theory (48), psychological need frustration derives from need-thwarting environments, and it can further contribute to psychological distress such as depression. Therefore, psychological need frustration might serve as a mediator of the link between peer relationship quality and depression.

The results of the current study implied that the serial mediating effect of the two mediators was significant. That is to say, phubbing behavior decreased the quality of peer relationships and then frustrated fundamental psychological needs; eventually, the severity of depression was enhanced. This serial mediating effect of peer relationship quality and psychological need frustration could be understood through the combination of Bandura’s triadic reciprocal determinism and the self-determination theory, which assume that personal factors are determined by behavioral and environmental factors, and psychological need frustration is the mediator of social contexts and mental illness (95). In the present study, phubbing and peer relationship quality were regarded as the behavioral and environmental determinants, respectively, and they sequentially contributed to the personal factors (psychological need frustration and subsequently depression). All this evidence not only confirmed the positive relationship between phubbing and the actors’ severity of depression but also elucidated underlying psychological mechanisms of peer relationship quality and psychological need frustration. Findings in this study also suggested that holistic interventions targeting both social relationships and psychological needs could be more effective in mitigating depression among adolescents who engage in phubbing. Such interventions could include group therapy to enhance peer connections alongside individual counseling to address psychological needs.

### 4.5 Gender differences in the serial mediation model

The results of the present study demonstrated the serial mediating effects of peer relationship quality and psychological needs frustration on the link between phubbing and depression were established in both male and female populations. The outcomes of the multi-group comparison analysis also indicated that there was no significant difference between the two gender groups, indicating that the path coefficients were similar for both genders. Although prior researchers provided mixed evidence concerning gender differences in these study variables or some links of the serial mediation model (see Section 1.5), this study demonstrated that the basic tendency from the antecedents to the consequences of the proposed model was comparable across genders. The results of this study were consistent with previous studies that showed that the study variables are comparable between male and female populations (59, 96–98), and gender is not a moderator of phubbing, smartphone addiction, or social exclusion and their downstream consequences (11, 99, 100). These evidences imply that both boys and girls would be equally influenced by their phubbing behaviors and the subsequent detrimental consequences. Theoretically, these results provided empirical supports for the cross-gender invariance of the assumptions of the basic psychological need theory and Bandura’s triadic reciprocal determinism (48, 95). And practically, these outcomes indicated that intervention measures targeting to relieve phubbing behaviors and depression, enhance peer relationship quality, and decrease psychological needs frustration should be put into effect regardless of the genders of the adolescents.

## 5 Implications

The outcomes of the present study provide several implications. At the micro level, this study shows that phubbing others may hurt oneself, and if an individual intends to have a good relationship with their peers and keep a healthy mind, putting down the smartphones and enjoying real life may be a good choice. At the macro level, the current study implies that the interpersonal surroundings are created by and will consequently influence everyone in them to some extent. Therefore, indulging in one’s behaviors may worsen interpersonal relationships and hurt oneself. At a policy level, these findings could inform the development of educational programs that promote healthy smartphone use and foster strong peer relationships in school settings. Such programs could be integrated into the curriculum to help adolescents develop the skills needed to navigate social interactions both online and offline. Future studies are encouraged to explore the occurrence and maintenance mechanisms of other unhealthy behaviors, which might be helpful for protecting adolescents from their detrimental consequences. Finally, to relieve the negative effects of phubbing on actors’ depression levels, measures should be taken to improve adolescents’ peer relationship quality and promote the healing of their psychological needs, meanwhile, gender stereotypes should be avoided.

## 6 Limitations and future research

The current study has some limitations. First, because this study uses a cross-sectional design, which limits the ability to infer causality from the findings, future researchers should conduct longitudinal studies to provide more evidence for the aforementioned relationships. Second, most of the participants in the current study are adolescents. However, prior studies have shown that there is a high prevalence of smartphone addiction among university students. Accordingly, future studies may examine the same relationship using a university student sample. Third, although participants are enrolled from different regions in China, the sample is essentially a convenience sample; future studies are encouraged to generalize the results of this study by using a probability sample or more randomized samples.

## 7 Conclusion

This study examined the link between phubbing and depression as well as the mediating effects of peer relationship quality and psychological need frustration. The results indicated that (1) phubbing behavior would be positively associated with depression among adolescents, (2) peer relationship quality would mediate this relationship, (3) psychological need frustration would also serve as a mediator, and (4) peer relationship quality and psychological need frustration sequentially would mediate the association between phubbing and depression. This study contributed to the growing body of literature on the psychological impacts of smartphone use by elucidating the mechanisms through which phubbing leads to depression. Future research should continue to explore these relationships in diverse populations and examine potential interventions that could mitigate the negative effects of phubbing on mental health.

## Data Availability

The raw data supporting the conclusions of this article will be made available by the authors, without undue reservation.
